# Luteal phase decrease in packed cell volume in healthy non‐pregnant and pregnant bitches

**DOI:** 10.1002/vms3.1195

**Published:** 2023-07-19

**Authors:** Rachel Moxon, Rebekah Dutton Worsfold, Julie Davis, Wendy Adams, Gary C. W. England

**Affiliations:** ^1^ Guide Dogs National Centre Leamington Spa UK; ^2^ School of Veterinary Medicine and Science University of Nottingham Sutton Bonington UK

**Keywords:** anaemia, dog, haematocrit, oestrus cycle, pregnancy

## Abstract

**Objectives:**

To establish packed cell volume (PCV) ranges for non‐pregnant, pregnant and post‐partum bitches from day 10 of proestrus, investigating any relationship with parity and litter size.

**Methods:**

This prospective cohort study used 37 healthy breeding bitches to examine PCV counts from routine blood samples collected every 4 weeks, from day 10 of proestrus, as part of routine PCV monitoring.

**Results:**

For pregnant (*n* = 19) and non‐pregnant (*n* = 18) bitches, PCV decreased until week 8 (corresponding to 8.5 ± 1.1 days before whelping for pregnant bitches) and recovered by 16–20 weeks after the initial sample; bitches that whelped average and large litters showed greater declines. PCV began to recover sooner for bitches that had previously whelped one or two litters compared to bitches that had previously whelped three or more litters. There was a significant three‐way interaction between time after the onset of proestrus, litter size and the number of previous litters which demonstrated that the large decrease in PCV for bitches that had previously whelped three or more litters only occurred in bitches that were expecting an average or large sized litter.

**Clinical Significance:**

Chronological variation in PCV for pregnant and non‐pregnant bitches was established during the reproductive cycle. There was no evidence to suggest that routine PCV measurement for normal, healthy bitches would be beneficial. However, knowledge from this study may be useful when deciding whether to prospectively monitor a bitch where there is a history of previous pregnancy‐related anaemia, when performing a caesarean section due to the anticipated blood loss during surgery, or when examining blood profiles for post‐litter bitches.

## Graphical abstract

Packed cell volume (PCV) for pregnant (*n* = 19) and non‐pregnant (*n* = 18) bitches measured every 4 weeks from day 10 of proestrus decreased until week 8 and recovered by 16–20 weeks after the initial sample; bitches that whelped average and large litters showed greater declines. There was a significant three‐way interaction between time, litter size and the number of previous litters; the large decrease in PCV for bitches that had previously whelped three or more litters only occurred in bitches that were expecting an average‐ or large‐sized litter. Knowledge from this study may be useful when deciding whether to prospectively monitor a bitch where there is a history of previous pregnancy‐related anaemia, when performing a caesarean section, or when examining blood profiles for post‐litter bitches.

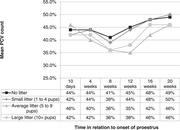

## 1 INTRODUCTION

The haematocrit (Hct) or packed cell volume (PCV) is essentially the percentage of blood volume which is occupied by the erythrocytes (Brockus, 2011). Measurement of Hct/PCV allows an understanding of oxygen‐carrying capacity of the blood and provides a simple estimation of how this may change in disease states. Conventionally, PCV was measured following centrifugation of a thin glass tube filled with blood, enabling the relative length of the tube containing red blood cells to be compared with the total length of tube containing blood, thus establishing the percentage PCV. This method is considered to be the gold standard, and is suggested to be accurate with a small inherent error of ±1% (Brockus, 2011; Gebretsadkan et al., 2015). More recently, automated blood cell counters have been developed to provide a calculation‐based method of establishing the Hct using the erythrocyte count and the mean corpuscular volume (Acker et al., 2012; Brockus, 2011). There are also newer methods of calculating the Hct by measuring electrical conductivity and using the principle that blood plasma is conductive, whereas the erythrocyte membrane is largely non‐conductive (broadly electrical conductivity is inversely related to Hct).

Each of the measurement methods are subject to their own potential technical errors, for example (1) inadequate centrifugation would result in erroneously high PCV, and operator error when filling the tube or placing the tube when reading results could affect results using the glass tube method; (2) cell counting errors would affect the calculation‐based Hct; and (3) errors in measurement of conductivity would affect electrical conductivity‐calculated Hct (Breheny et al., 2019; Gebretsadkan et al., 2015; Powell & Torrence, 2012; Tvedten, 1993). Physiological or pathological changes of the sample may differentially affect accuracy of the three methods; agglutination of erythrocytes would not alter the glass tube PCV or electrical conductivity‐calculated Hct but would falsely decrease cell counting calculation‐based Hct. Conversely, changes in plasma constitution, such as in osmolality as described by Watson and Maughan (2014), may change electrical conductivity and falsely change electrical conductivity‐calculated Hct, but not impact glass tube PCV or cell counting calculation‐based Hct.

In the literature, there are some good examples of differences in methodology affecting the PCV/Hct result. Studies using blood from many species including humans, chelonians, white rhino, monkeys, horses, dogs, cats and rats have shown both under‐ and over‐estimation of Hct from automated analysers in comparison to PCV using the manual microhaematocrit method, although the two measures are often strongly positively correlated (Becker et al., 2008; Gebretsadkan et al., 2015; Pastor et al., 1997; 1998; Roleff et al., 2007; Streyrer et al., 2022; Tabata et al., 1998; Wong & Girolamo, 2021). Becker et al. (2008) suggested the need for analyser‐specific Hct reference intervals to avoid clinically relevant misinterpretation. The use of the terms PCV and Hct within the literature is also confusing, with some authors who measure PCV using a microhaematocrit method stating results for Hct (Dimço et al. 2013; De Cramer et al. 2016; Günzel‐Apel et al. 1997). Unfortunately, a number of publications define normal ranges without clearly defining the methodology used for measurement of PCV/Hct.

In dogs, there are variations in reported normal values for PCV/Hct, with reference ranges varying between laboratories. Lower values for normal PCV between 35 and 42, and for Hct between 37 and 42, and upper normal values between 54 and 57 for PCV and between 55 and 62 for Hct have been proposed (see Table S1). Some of this variation may relate to the study population used to establish the normal range, the laboratory and the technical method used to measure the PCV/Hct.

During late pregnancy, an increase in plasma volume is suggested to cause haemodilution and contribute to decreased PCV in bitches (Ajala et al., 2011; Concannon, 2002; Concannon & Lein, 1989; Chaudhari & Mshelia, 2006; Kaneko et al., 1993; Klainbart et al., 2017; Verstegen‐Onclin & Verstegen, 2008) as well as in other animal species, including sows (Tumbleson et al., 1970), goats (Azab & Abdel‐Maksoud, 1999; Mbassa & Poulsen, 1991) and humans (Darby et al., 1953; De Benoist et al., 2008). The reported extent of the decline in PCV associated with pregnancy in bitches varies between studies of which several have not serially measured PCV/Hct and in which different methods of PCV/Hct measurement are used which could impact the results. There are also large ranges in PCV/Hct values for pregnant bitches, irrespective of sampling time, which demonstrate individual variation among bitches (see Table S2). In studies which have demonstrated a decline in PCV/Hct during pregnancy, or a difference in values between pregnant and non‐pregnant bitches in late‐stage pregnancy, mean PCV/Hct of between 30% and 41% has been described, although mean values are not always reported to fall below the normal reference range (Ajala et al., 2011; Concannon et al., 1977; Chaudhari & Mshelia, 2006; Dimço et al., 2013; Günzel‐Apel et al., 1997; Kaneko et al., 1993; Klainbart et al., 2017; Mshelia et al., 2005; Tietz et al., 1967). Where a decline in PCV/Hct is detected, it is reported to have returned to normal levels by 8–12 weeks post‐partum (Concannon, 2002; Günzel‐Apel et al., 1997). Interestingly, in parturient bitches, PCV/Hct may increase. De Cramer et al. (2016) reported mean PCV of 44.2% (95% confidence interval [CI] = 43.8%–44.6%) for healthy bitches immediately before 406 elective caesarean surgeries and found values within the normal range, while Frehner et al. (2018) reported mean Hct of 45.3% ± 5.9% for 22 parturient bitches presented for dystocia, with Hct greater than 37% for 19 bitches, suggesting that there might be haemoconcentration and/or other mechanisms in place that result in higher values at parturition. It was hoped that by placing prospective results in the framework of a critical appraisal of the literature, clarity could be bought to this subject area where there has previously been significant variation in observations.

In this study, haematology was prospectively monitored as part of routine breeding management. The purpose was to examine PCV in non‐pregnant, pregnant and post‐partum bitches using serial blood collections throughout oestrus, pregnancy and lactation, and examine any relation to parity and litter size. The authors hypothesize that, in healthy bitches in this well‐managed breeding programme, PCV will decline during pregnancy and the extent of the decline will be related to litter size and parity.

## 2 MATERIALS AND METHODS

Ethical approval for this prospective cohort study was received from Guide Dogs’ Veterinary Advisors Committee. Data were gathered from electronic health records held for bitches in a large breeding colony that had been subject to routine PCV monitoring from day 10 of proestrus for 20 weeks. Six serial blood samples were collected from 37 normal, healthy, large breed bitches as part of a routine health programme of PCV monitoring (one German Shepherd, five Golden Retrievers, two Golden Retriever cross Labradors and 29 Labradors aged between 1.4 and 6.8 years of age on day of first sample collection). Bitches were in their mating (*n* = 21) or non‐mating season (*n* = 16). All bitches were housed in volunteer homes with the exception of a 21‐day period from day 1 of proestrus when they were housed in a breeding kennel facility. Bitches were fed high‐quality dry diets and received regular health checks from qualified staff performed every 4 months increasing to weekly during pregnancy and lactation. The first blood samples were collected on approximately day 10 from the start of proestrus and then continued approximately every 4 weeks until the sixth sample at 20 weeks. Two bitches that were mated failed to conceive, therefore data were for 19 pregnant (mean age = 3.9 ± 0.4 years) and 18 non‐pregnant (mean age = 4.1 ± 0.4 years) bitches. Age (Mann–Whitney *U* = 184, *p* = 0.707) was not different between the groups, and breed distribution was similar between pregnant and non‐pregnant bitches; statistical analysis was not possible due to low numbers of bitches of breeds other than Labradors. For pregnant bitches, three PCV samples were collected pre‐partum and three post‐partum; the final pregnant sample was 8.5 (mean ± 1.1) days before whelping. Blood samples were collected from the cephalic vein using a 5‐mL syringe and 21‐gauge needle into EDTA tubes. A small amount of blood was immediately transferred into a plugged Hct tube, placed in a Dyaset Mini Hematocrit Centrifuge and spun at 11,000 rpm for 3 min. PCV was measured on the inbuilt reader within the machine and was recorded. All sample collection and measurements were conducted by one of the authors.

### 2.1 Quantitative variables and statistical analyses

Data were presented as mean ± SEM (minimum, maximum). All statistical analyses were performed using SPSS (IBM SPSS Statistics for Windows, Version 22.0. Armonk, NY: IBM Corp). Wilcoxon paired tests were used to examine differences in day 10 and 8‐week PCV for pregnant and non‐pregnant bitches. Two three‐way repeated‐measures analyses of variance (ANOVAs) were used to assess whether the size of the litter (if any) and the number of previous pregnancies significantly affected PCV in the 20 weeks following day 10 of proestrus. In both ANOVAs, the within‐groups independent variable was time after the onset of proestrus (PCV was measured at six time points—day 10 of proestrus, then 4 weeks, 8 weeks, 12 weeks, 16 weeks and 20 weeks from day 10 of proestrus), while the between‐groups independent variables were breed, the size of the litter (four groups; no litter, small litter, average litter and large litter) and the number of previous pregnancies (four groups; no previous litters, one previous litter, two previous litters and three or more previous litters). In the first three‐way ANOVA, litter size groupings of small (less than five pups, *n* = 3), average (five to nine pups, *n* = 12) and large (more than nine pups, *n* = 4) were used for the litter size variable, as it was felt that these groupings had the best external validity. In the second ANOVA, the definitions of small, average and large litter sizes were adjusted to produce three groups that were more equally sized while retaining 7 as the median value (small: less than six pups, *n* = 4; average: six to eight pups, *n* = 7; large: more than eight pups, *n* = 8; see Figure S1). This was to ensure that any significant effect dependent upon litter size was not obscured by small base sizes. The group sizes for the number of previous litters were more evenly distributed (no previous litters = 8, one previous litter = 9, two previous litters = 10, three or more previous litters = 10; Figure S2). Effects were considered statistically significant if *p* < 0.05. Tukey's HSD test was used to assess which differences between the litter size groups were contributing to the significant main effect of litter size. To determine the extent of the effect of litter size on PCV, a linear regression model was used with three independent variables (litter size, number of previous litters and time).

## 3 RESULTS

**FIGURE 1:**
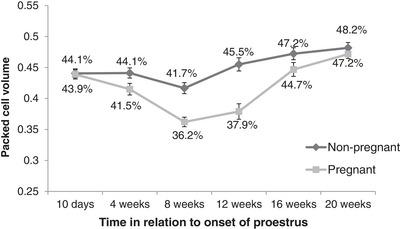
Mean PCV (%) (±SEM) for 19 pregnant and 18 non‐pregnant bitches measured at 4‐week intervals from day 10 of proestrus. Samples at 8 weeks represented the final pre‐partum sample for pregnant bitches at a mean of 8.5 ± 1.1 days before whelping.

During the study period, no clinically significant anaemia requiring treatment developed in any bitch. PCV decreased in both pregnant (*p* < 0.001) and non‐pregnant (*p* = 0.027) bitches from 43.9% ± 0.8% (38%–49%) and 44.1% ± 0.7% (40%–51%), respectively, at day 10 to 36.2% ± 0.8% (30%–43%) and 41.7% ± 0.9% (33 to 46%), respectively, at 8 weeks after the initial sample (the final pre‐partum PCV measurement for pregnant bitches). At 8 weeks after the initial sample, 10 out of 19 pregnant and two out of 18 non‐pregnant bitches had PCV lower than 37%. By 20 weeks, PCV recovered to higher than measured at the first proestrus sample interval for both pregnant and non‐pregnant bitches (Figure 1). No significant correlation was found between litter size and previous litters for either the first (*r* = –0.158, *p* = 0.349) or second (*r* = –0.218, *p* = 0.195) litter size groupings.

The first three‐way repeated‐measures ANOVA found significant main effects of time after the onset of proestrus (*F*(5,125) = 31.829, *p* < 0.001) and litter size (*F*(3,25) = 5.707, *p* = 0.004), and significant interactions between time after the onset of proestrus and litter size (*F*(15,125) = 4.403, *p* < 0.001), time after the onset of proestrus and previous litters (*F*(15,125) = 2.297, *p* = 0.007) and time after the onset of proestrus, litter size and previous litters (*F*(25,125) = 1.921, *p* = 0.010). There was no significant main effect of the number of previous litters, and no significant interaction between litter size and previous litters. There was no significant main effect of breed and no significant interactions involving breed. The second three‐way repeated‐measures ANOVA found the same pattern of significance and therefore all subsequent results relate to the first significance test.

### 3.1 The significant main effects

**FIGURE 2:**
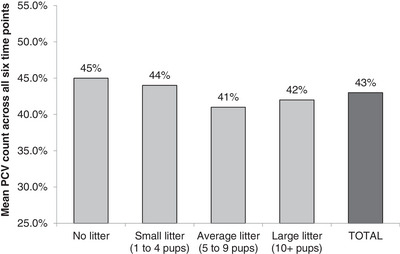
The mean PCV (%) for each of the litter size groups across the six time points (10 days, 4 weeks, 8 weeks, 12 weeks, 16 weeks and 20 weeks). The combined total for all 37 bitches is also shown.

PCV was lower in bitches with an average‐sized litter (mean = 41%) relative to the non‐pregnant bitches (mean = 45%, *p* = 0.008), although the lowered PCV in bitches with a large litter (mean = 42%) relative to the non‐pregnant bitches almost achieved significance (*p* = 0.055; Figure 2). Regardless of litter size and previous pregnancies, PCV decreased in the 4 and 8 weeks following day 10 of proestrus and then recovered over the next 12 weeks.

### 3.2 The significant two‐way interactions

**FIGURE 3:**
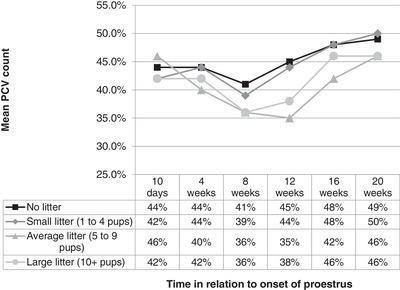
Mean PCV (%) for non‐pregnant bitches and bitches that whelped small (*n* = 3), average (*n* = 12) and large litters (*n* = 4).

Although PCV for non‐pregnant bitches decreased significantly, at week 8, the decrease was larger for pregnant bitches; the non‐pregnant PCV values remained higher or equal to the pregnant ones during the subsequent weeks. The bitches that whelped average and large sized litters showed greater declines in PCV than the other two groups across the whole length of the study (Figure 3).

**FIGURE 4:**
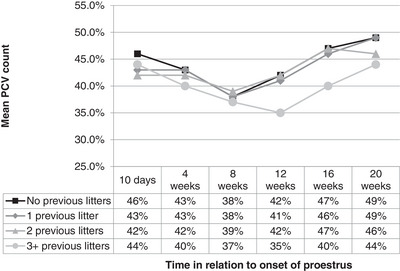
Mean PCV (%) for bitches that had no (*n* = 8), one (*n* = 9), two (n = 10) or three or more (*n* = 10) previous litters.

For the first 8 weeks, PCV for the four groups was similar. Although the PCV for bitches that had previously whelped two or fewer litters began to increase after 8 weeks, PCV for bitches that had whelped three or more previous litters declined for longer. For this group, PCV took an extra 4 weeks before starting to increase; thereafter, the increase was at a similar rate as in the other groups, although PCV had not returned to initial values by the time the study ended at 20 weeks (Figure 4).

### 3.3 The significant three‐way interaction

**FIGURE 5:**
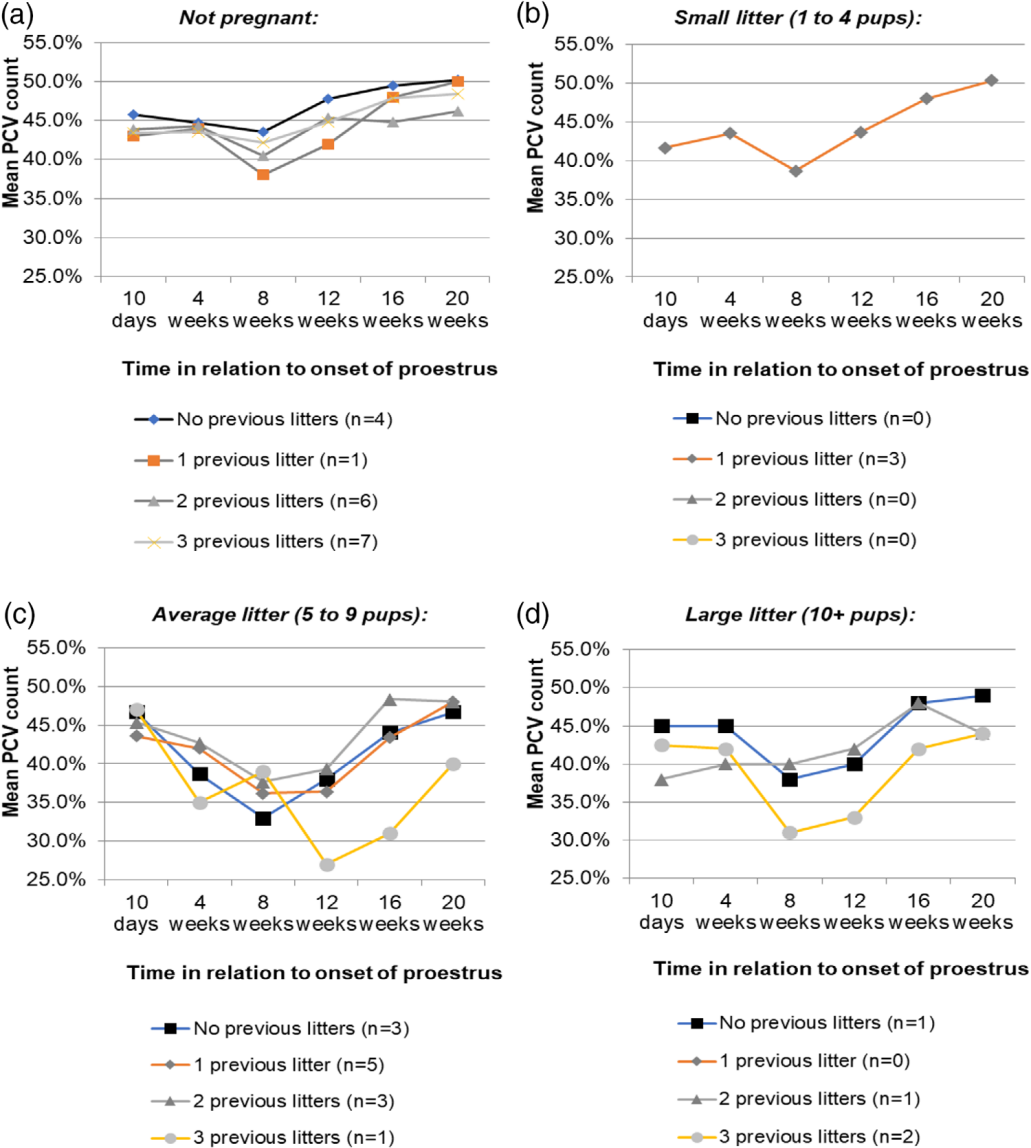
The negative impact on PCV (%) of having had three or more litters in the past only appeared in bitches that were expecting average or large‐sized litters.

Investigation of the significant three‐way interaction between time after the onset of proestrus, litter size and the number of previous litters demonstrated that the large decrease in PCV in bitches that had three or more previous litters only occurred in those with a current average or large litter (Figure 5). No differences were observed in the small litter group, which only represented one of the four possible combinations (i.e., small litter plus one previous litter).

The regression equation for the effect of litter size was PCV = 0.432 – 0.004 (Pups whelped) – 0.006 (Previous litters) + 0.009 (Time). Therefore, for each puppy increase in litter size, PCV declined by 0.4% (*r* ^2^ = 0.206, *p* < 0.001).

## 4 DISCUSSION

This study used a centrifugation method to measure PCV and reported the changes in PCV for non‐pregnant, pregnant and post‐partum bitches for a 20‐week period commencing 10 days from the start of proestrus. In addition, effects of litter size and parity on PCV were identified.

PCV for both pregnant and non‐pregnant bitches declined in the 8 weeks following the onset of proestrus, though the decline was greater for pregnant bitches. This is consistent with findings reported by other authors (Günzel‐Apel et al., 1997; Tietz et al., 1967). Ajala et al. (2011) did not observe a decrease in PCV in two pseudopregnant bitches (PCV remained >50%) compared to six pregnant bitches and suggested, therefore, that pregnancy and pseudopregnancy can be distinguished using these measurements. However, variation in normal PCV/Hct values reported in the literature and the decline in PCV reported in pregnant and non‐pregnant bitches in the present study, and by others, contradict this suggestion. There is little evidence that PCV/Hct measurement can be used to determine pregnancy status in the bitch. Tietz et al. (1967) proposed that the decrease in PCV may be instigated by the mechanisms that initiate oestrus and may help with the rapid change in blood volume immediately following parturition caused by the rapid decrease in vascular space. However, recent findings suggest high PCV values at parturition, both in healthy bitches presented for elective caesarean and in bitches with dystocia. Perhaps such an increase may be in preparation for the effects of events relating to parturition, such as placental separation, which may result in blood loss. Further research regarding PCV in the 48 h prior to parturition may help clarify some of the current findings. The decrease in PCV for pregnant and non‐pregnant bitches may strengthen the argument that haemodilution during gestation is not the sole contributing factor to PCV decline (Nivy et al., 2019).

Relationships between PCV and litter size, breed and age have been proposed, with lower PCV/Hct reported for bitches expecting larger litters, bulldogs and younger bitches (De Cramer et al., 2016; Kaneko et al., 1993; Mshelia et al., 2005). The magnitude of decline in PCV/Hct reported by different authors in relation to increasing litter size has varied (0.14%–1.1% decrease in PCV/Hct for each one pup increase in litter size [De Cramer et al. 2016; Kaneko et al. 1993]). Kaneko et al. (1993) suggest that variation in PCV reported in different studies may be caused by litter size differences between study populations; indeed, litter size is commonly not reported or investigated. Within the current study, the degree and duration of the decline in PCV over time were affected by pregnancy, litter size (especially bitches that were pregnant with average or large sized litters) and the number of previous pregnancies (specifically those that had three or more previous litters). An effect of parity on decrease in PCV has previously been reported in goats (Mbassa & Poulsen, 1991) but, to the authors’ knowledge, has not previously been reported in bitches. A parity effect on serum cobalamin concentrations was reported by Nivy et al. (2019). Mshelia et al. (2005) reported lower PCV values for bitches aged over 3 years; however, data for previous pregnancies were not reported or investigated. The effect of litter size is likely to be influenced by increased haemodilution and plasma volume with increasing litter size, and subsequently decreasing PCV; future investigations could include total protein measurements to confirm the contribution of that process, but the mechanism of influence of previous pregnancies independent of any effect of age is unclear.

PCV then increased over the subsequent 12 weeks which resulted in values that were higher at the 20‐week sample than proestrus. The 20‐week sample represents the end of metestrus/start of anoestrous. Anoestrus PCV/Hct values within the literature have been reported to be higher than at other stages by some authors (Chitrang et al., 2019; Günzel‐Apel et al., 1997; Willson et al., 2012). The increase to anoestrus PCV was also impacted by litter size and previous pregnancies. Final PCV values were lowest for bitches that had two or more previous litters and were not pregnant or pregnant with large litters during the study. One bitch with an average‐sized litter that had three or more previous pregnancies was also slower to demonstrate an increase in PCV, with PCV slightly higher than 30% at the 16‐week sample and with lower final values. These findings are interesting as they suggest that the lower PCV associated with pregnancy persists through dioestrus, and demonstrate the importance of considering a recent pregnancy and litter for a bitch presenting with low PCV values on haematological examination.

The pathophysiology of pregnancy‐related anaemia in the bitch has not been well studied. Iron deficiency has been reported to increase as pregnancy progresses in women (Goonewardene et al., 2012; Mei et al., 2011). However, Nivy et al. (2019) unexpectedly reported higher total iron‐binding capacity and serum iron concentrations in late than mid‐pregnancy in 48 pregnant bitches. Interestingly, while suggesting that there was no association with pregnancy‐related anaemia, Nivy et al. (2019) reported significantly lower values for serum cobalamin and higher levels of hypocobalaminaemia for bitches at 4 days prior to parturition compared to mid‐pregnancy. Cobalamin was also found to be affected by parity and litter size, with cobalamin concentrations decreasing with increasing parity and litter size. Combined with our findings regarding litter size, previous pregnancies and PCV, further investigation of the relationship between blood mineral content and potential deficiencies alongside normal haematology including PCV for bitches during pregnancy could be useful to examine these hypothetical associations in more detail.

The reason for a similar, albeit smaller decrease in PCV observed in non‐pregnant bitches is less clear and worthy of further consideration. PCV may be influenced by reproductive hormone‐mediated effects on vitamin and mineral transport or binding. In pregnant and non‐pregnant bitches, progesterone remains high for approximately 2 months post‐ovulation (Concannon et al., 1975; Verstegen‐Onclin & Verstegen, 2008). While serum plasma progesterone concentrations are similar for both pregnant and non‐pregnant bitches, progesterone production and metabolism are suggested to be higher in pregnant bitches (Concannon, 2011; Gudermuth et al., 1998; Holst et al., 2019), although it does not seem that the amount of circulating progesterone is related to litter size. Klainbart et al. (2017) showed there was no relation between haematology parameters and serum progesterone concentration; however, the authors suggest that haemostasis may be impacted by progesterone metabolites in conjunction with other hormones and their metabolites. Pregnant bitches likely have higher concentrations of progesterone metabolites which may explain to some degree the haematological differences observed between pregnant and non‐pregnant groups.

The decline in PCV reported here in healthy bitches with normal, spontaneous onset of parturition is similar to the decline in Hct documented by Nivy et al. (2019) where at late pregnancy 77% of bitches were reported to be anaemic and mean Hct was 36% (interquartile range [IQR] = 32% to 39%), while approximately 4 weeks previously mean Hct was 40% (IQR = 37%–43%, PCV of 41.5% for pregnant bitches in our study at a similar time). However, this seems to conflict with the findings of De Cramer et al. (2016) who described a mean PCV of 44% (95% CI = 43.8%–44.6%) in pregnant bitches immediately before 406 caesarean surgeries and PCV lower than 37% in only 10 (2.5%) of these cases. The authors suggested that the pregnancy‐related anaemia described by others may not occur in well‐managed bitches with good nutrition and veterinary care, and that anaemia during pregnancy may be caused by other factors rather than pregnancy itself. However, De Cramer at al. (2016) did not collect serial blood samples to measure PCV throughout oestrus and pregnancy; therefore, whether PCV declined to 44% or remained constant at 44% is unknown. Frehner et al. (2018) also reported Hct values within the normal reference range (>37%, mean 45.3% ± 5.9%) for 19 out of 22 bitches presented at a small animal clinic for dystocia management, while our work reports PCV lower than 37% in 10 out of 19 bitches (mean PCV was 36%) at an average of 8 days before whelping. Given the similarity in mean PCV/Hct values at 4–8 days prior to parturition between this study and the work of Nivy et al. (2019), and low PCV/Hct in late pregnancy reported by others, it is possible that there are physiological changes immediately prior to parturition that may cause elevation of PCV/Hct at this time. It is also possible that changes in the bitch's behaviour, including reduced food and water consumption and heavy panting, may cause haemoconcentration at the time of parturition, leading to conflicting results from many studies. A sample at the time of partition was not collected within the present study.

In terms of clinical significance, it is important to recognise that the values of PCV/Hct in late pregnancy may be of great importance. Although in our study these animals had PCV values of 35% and might be described as having a mild anaemia, a proportion of bitches may suffer dystocia and require caesarean operation which may result in further potential blood loss and has been shown to potentially reduce PCV by nearly 7% (De Cramer et al., 2016). Such a further reduction in PCV would be considered as a moderate anaemia that could be clinically significant and knowledge from this study may be useful when performing a caesarean section due to the anticipated blood loss during surgery.

Within the present study, bitches were from a well‐managed breeding programme for assistance dogs, were all fed high‐quality dry diets and their health was regularly monitored, apparently disputing the suggestion of De Cramer et al. (2016). It is possible that differences between studies may be influenced by multiple factors including bitch breed, the method of PCV/Hct measurement (Frehner et al., 2018), mineral status (Nivy et al., 2019) as well as by reproductive history and expected litter size at the current pregnancy. Repeated serial measurements over several pregnancies for a large number of individual bitches would provide interesting data on individual bitch influence on changes in PCV. These could include bitches of different ages; age was not considered within the current study due to the relationship with parity. Additionally, repeat measurement of samples using different methods for PCV/Hct measurement would be useful in increasing understanding related to the differences between manual and automated methods, which could aid in interpretation of apparently conflicting information from many studies.

The relatively large number of between‐groups variable levels in these analyses means that the sample was spread thinly across them. There were no bitches with small litters that had previously had two or more litters or that had not had a litter and there were low numbers of bitches that had large litters across all previous litter groups. This prevented the study from being able to ascertain whether the negative impact of having whelped three or more litters in the past applied to the pregnant bitches that whelped a small litter in the same way that it applied to those that whelped larger litters. The low base size does not affect the validity of the significance testing (ANOVAs take base sizes into account), but it makes interpreting the interactions more difficult than with a larger sample. It would be useful to repeat the study with data from a larger number of bitches that are more equally distributed between the variable groups to cover all of the 16 litter size–previous litters combinations. Additionally, only PCV and not full blood profiles were  analysed for bitches in the current study. In future studies, it would be useful to include other factors which may impact PCV such as complete blood count data.

In summary, PCV declined for both pregnant and non‐pregnant bitches over the 20‐week period, suggesting that haemodilution associated with pregnancy is one of many contributing factors for PCV decline. The decline was greater for pregnant bitches and the data support the occurrence of a pregnancy‐related anaemia in the bitch. Litter size and time after the onset of proestrus affected PCV, and there were statistically significant interactions between time after the onset of proestrus and litter size, time after the onset of proestrus and the number of previous litters, and time after the onset of proestrus, litter size and the number of previous litters. The magnitude and duration of the decline in PCV were more remarkable for bitches that had larger litters and that had three or more previous litters.

## AUTHOR CONTRIBUTIONS

**Rachel Moxon**: Conceptualization; investigation; supervision; writing—original draft; writing—review and editing. **Rebekah Dutton Worsfold**: Formal analysis; software; writing—review and editing. **Julie Davis**: Data curation; investigation; project administration; resources; writing—review and editing. **Wendy Adams**: Conceptualization; data curation; investigation; methodology; project administration; supervision; writing—review and editing. **Gary England**: Conceptualization; investigation; methodology; supervision; writing—original draft; writing—review and editing.

## CONFLICT OF INTEREST STATEMENT

The authors declare no conflicts of interest.

### ETHICS STATEMENT

Ethical approval for this prospective cohort study was received from Guide Dogs' Veterinary Advisors Committee.

### PEER REVIEW

The peer review history for this article is available at https://publons.com/publon/10.1002/vms3.1195.

## Supporting information

Supporting Information

Supporting Information

Supporting Information

Supporting Information

## Data Availability

Guide Dogs encourages high‐quality research to improve the health, temperament and welfare of its dogs. Given the unique nature of this population and the data we collect, Guide Dogs is keen to ensure the integrity of any associated research and prevent misrepresentation. As such, we do not routinely release raw data but will allow its use within high‐quality research proposals that have been approved under Guide Dogs governance process.
