# Supplementary figures and images for: Luteal phase decrease in packed cell volume in healthy non‐pregnant and pregnant bitches
Source: Vet Med Sci. 2023 Jul 19;9(5):1989–97. doi: 10.1002/vms3.1195 (PMC10508517; doi:10.1002/vms3.1195)

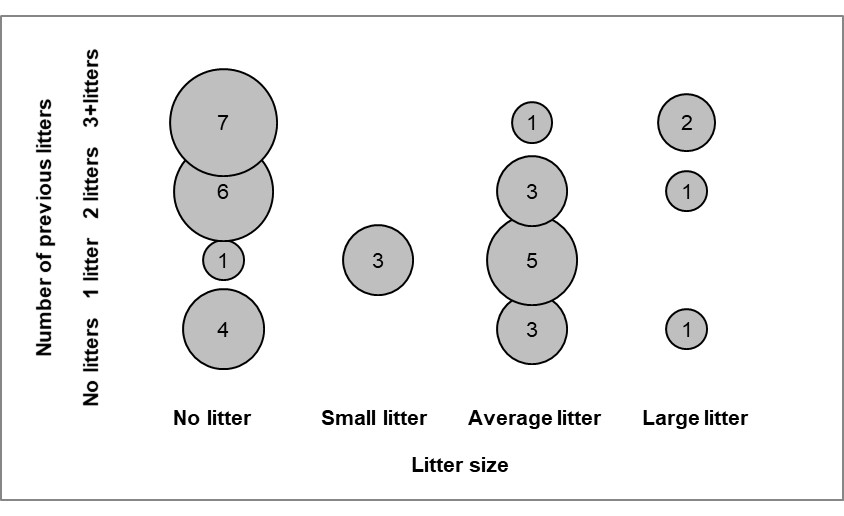

Supplement: Supplementary file 1 — Supporting Information [file VMS3-9-1989-s002.jpg]

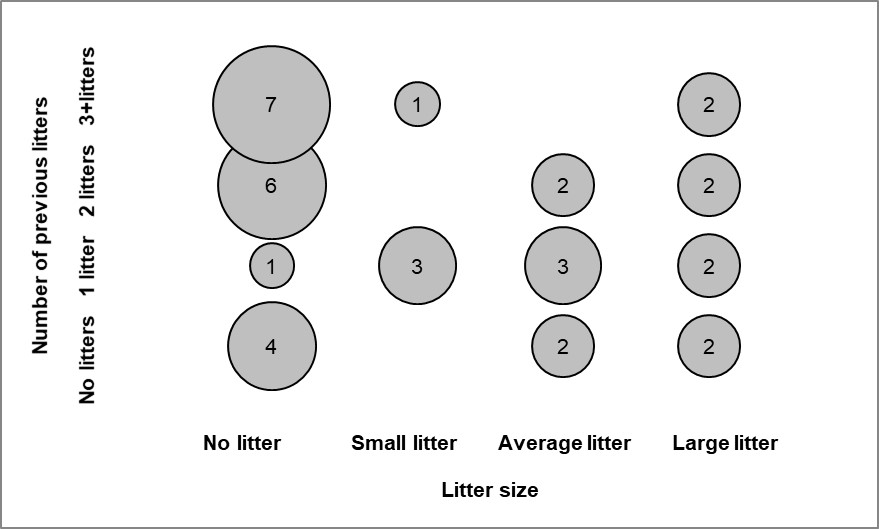

Supplement: Supplementary file 2 — Supporting Information [file VMS3-9-1989-s001.jpg]
