# Supplementary material for: Luteal phase decrease in packed cell volume in healthy non‐pregnant and pregnant bitches
Source: Vet Med Sci. 2023 Jul 19;9(5):1989–97. doi: 10.1002/vms3.1195 (PMC10508517; doi:10.1002/vms3.1195)
Supplement: Supplementary file 3 — Supporting Information [file VMS3-9-1989-s003.docx]

Supplementary information

Table 1. The normal reference ranges suggested for PCV/Hct for dogs from different sources. Accessed 27 January 2023.

| Source | Hct | PCV |
| --- | --- | --- |
| Cornell University Animal Health Diagnostic Center^1^ | 41-58 | 42-54 |
| MSD Veterinary Manual^2^ | - | 35-57 |
| UC Davis^3^ | 40-55 | - |
| IDEXX^4^ | 37.3-61.7 | - |
| Vetnurse.co.uk^5^ | - | 37-55 |
| Moritz et al.^6^ | 42-62* |  |
| University of Guelph Animal Health Laboratory^7^ | 39-56 | - |
| Iowa State University Clinical Pathology Laboratory^8^ | 37-55 | - |
| Zoetis^9^ | 37-55 | - |
| BSAVA Manual of Canine and Feline Haematology and Transfusion Medicine^10^ | 40-56 | - |

*based on 2.5^th^ to 97.5^th^ percentiles

1. Cornell University Animal Health Diagnostic Center. Hematology (Advia 2120) Routine Hemogram Reference Intervals. https://www.vet.cornell.edu/animal-health-diagnostic-center/laboratories/clinical-pathology/reference-intervals/hematology

2. MSD Veterinary Manual. Hematology Reference Ranges. https://www.msdvetmanual.com/special-subjects/reference-guides/hematology-reference-ranges

3. UC Davis. Clinical Diagnostic Laboratory CBC Reference Intervals https://www.vetmed.ucdavis.edu/sites/g/files/dgvnsk491/files/local_resources/pdfs/lab_pdfs/UC_Davis_VMTH_Hematology_Reference_Intervals.pdf

4. Idexx. Reference Intervals for the IDEXX ProCyte Dx* Hematology Analyzer

https://www.idexx.com/media/filer_public/e2/16/e216b67b-2da8-4bd2-ad27-f75bafb7ed85/procyte-dx-reference-ranges-en.pdf

5. VetNurse.co.uk. PCV Measurement - Practical Task.

https://www.vetnurse.co.uk/nursing/w/vet-nurse-revision-2/585/pcv-measurement-practical-task

6. Moritz, A., Fickenscher, Y., Meyer, K., Failing, K. and Weiss, D.J. (2004). Canine and feline hematology reference values for the ADVIA 120 hematology system. Veterinary Clinical Pathology. 33, 32-38.

7. University of Guelph Animal Health Laboratory. Hematology reference intervals. https://www.uoguelph.ca/ahl/content/hematology-reference-intervals

8. Iowa State University Clinical Pathology Laboratory. Reference Intervals. https://vetmed.iastate.edu/vpath/services/diagnostic-services/clinical-pathology/testing-and-fees/reference-intervals

9. Zoetis. VETSCAN® HM5 Reference Ranges (Common Species) https://www2.zoetisus.com/content/_assets/docs/Diagnostics/technical-papers/HM5-Reference-Ranges-iPad-VTS-00426.pdf

10. BSAVA Manual of Canine and Feline Haematology and Transfusion Medicine. 2012. Appendix 1. Reference values for haematology and haemostasis. 2^nd^ Edn. Eds: Day, M. J. and Kohn, B. British Small Animal Veterinary Association, Gloucester, UK. Pp. 324
