# Supplementary material for: Luteal phase decrease in packed cell volume in healthy non‐pregnant and pregnant bitches
Source: Vet Med Sci. 2023 Jul 19;9(5):1989–97. doi: 10.1002/vms3.1195 (PMC10508517; doi:10.1002/vms3.1195)
Supplement: Supplementary file 4 — Supporting Information [file VMS3-9-1989-s004.docx]

Supplementary information

Table 2. The average PCV / Hct (%) reported in published studies measured at different time points in the oestrous cycle for pregnant (P) and non-pregnant (NP) bitches along with details of the bitches studied, sample intervals, method of measurement and whether a decline was observed for each group. Values reported for dioestrus are shown in the last few weeks of pregnancy column.

| Author and date | Study population | Sample interval | Method of measurement | Normal reference range cited | Descriptive statistics reported | Reported a decline during pregnancy | Reported a decline in non-pregnant bitches | Average PCV / haematocrit values reported for bitches (%) | | | | | |
| --- | --- | --- | --- | --- | --- | --- | --- | --- | --- | --- | --- | --- | --- |
|  |  |  |  |  |  |  |  | Anoestrous | Proestrus / oestrus | Approximately 4 weeks later | Last few weeks of pregnancy (approximately 8 weeks later) | Parturient | Two weeks after parturition |
| Chitrang et al., 2019 | 86 non-pregnant Beagle bitches | One measurement at each stage  Immature (8.4%)  Anoestrus (26.5%)  Proestrus (26.5%)  Oestrus (4.8%)  Dioestrus (33.7%) | Haematocrit  Automated haematology analyser using volumetric impedance and optical detection | None stated | Mean ± S.D | N/A | N/A | 45.1±5.2 | 40.0±2.0 to 43.6±4.1 | - | 42.7±5.4 (dioestrus) | - | - |
| Nivy et al., 2019 | 48 pregnant bitches from nine breeds | Mid and late pregnancy | Haematocrit ADVIA 2120 hematology system: flow cytometry peroxidase methodology | 37-57 | Median and interquartile range | Yes | N/A | - | - | 40.0 (37.0 to 43.0) | 36.0 (32.0 to 39.0) | - | - |
| Frehner et al., 2018 | 22 pregnant bitches of varying breeds presented with dystocia | One measurement before medication was administered | Haematocrit RAPIDpoint 500 blood gas system / Sysmex XT 2000 electrical conductivity (for 12 dogs | 37-61 | Mean ± S.D and range | N/A | N/A | - | - | - | - | 45.3±5.9 (35.0 to 55.0) | - |
| Klainbart et al., 2017‡ | 37 bitches, breed not stated | Proestrus/oestrus (n=17)  4^th^ week of pregnancy (n=10)  Last week of pregnancy (n=10)  Dioestrus (NP, n=10)  Anoestrus (n=10) | Haematocrit Advia 120 | 37-56 | Median and range | Yes | N/A | 42.5 (37.5 to 53.7) | 47.2 (37.6 to 57.1) | 41.7 (P) (32.0 to 49.6) | 39.9 (P) (31.4 to 46.3)  42.2 (NP) (37.6 to 47.5) | - | - |
| Klainbart et al., 2017 | 10 pregnant bitches, breed not stated | Three measurements 1) oestrus, 2)  4^th^ week of pregnancy, 3)  last week of pregnancy | PCV  Method not reported | 37-55 | Median and range | Yes | N/A | - | 45.5 (35.0 to 50.0) | 39.5 (32.0 to 46.0) | 39.0 (31.0 to 45.0) | - | - |
| De Cramer et al., 2016 | 324 pregnant bitches of varying breeds at 406 caesarean surgeries | One measurement before caesarean surgery | PCV  Microhaematocrit  capillary tube | 37-55 | Mean and 95% CI | N/A | N/A | - | - | - | - | 44.2 (43.8 to 44.6) | - |
| Dimço et al., 2013 | 16 pregnant and 16 non-pregnant bitches, breed not stated | One measurement in the second month of pregnancy | Haematocrit Microhaematocrit  capillary tube | 37-55 | Mean ± S.D | N/A | N/A | - | - | - | 41.0±4.9 (P)  45.4±3.6 (NP) | - | - |
| Willson et al., 2012 | 38 dioestrus Beagles and 48 Beagle bitches at other stages of the oestrous cycle | One measurement per dog | Haematocrit ADVIA 2120 hematology system: flow cytometry peroxidase methodology | None stated | Mean ± one S.E | N/A | N/A | 50.0±1.0 ¶ | - | - | 46.0±1.0 (dioestrus) |  | - |
| Ajala et al., 2011 | Six pregnant and two pseudopregnant small breed (10 to 12kg) bitches | Before mating  1 to 20 days after mating  21 to 42 days after mating (data not included here)  43 days after mating to parturition | PCV  Method not reported | None stated | Mean ± SEM | Yes | No | - | 51.4±0.9 (P and NP) | 42.3±7.5 (P)  50.9±0.5(NP) | 34.0±8.0 (P)  50.8±0.8 (NP) | - | 30.8±8.3 (P) |
| Kimberely et al., 2006^†^ | 10 pregnant bitches, breed not stated | Weekly samples from day of mating to parturition | PCV  Methods not stated in abstract | Not stated in abstract | Values not presented | Yes | N/A | - | - | - | ^†^Slightly below normal adult values | - | - |
| Mshelia et al., 2005 [18];  Chaudhari and Mshelia, 2006 | 39 mongrel bitches | One sample from a bitch at each stage  Immature (n=8)  Anoestrus (n=5)  Proestrus (n=5)  Oestrus (n=8)  Pregnant (n=7)  Dioestrus (NP, n=6) | PCV  Microhaematocrit | None stated | Mean ± S.D | N/A | N/A | 38.6±2.9 | 40.3±2.1 to 41.4±4.2 | - | 37.7±3.8§ (P)  40.2±2.8 (NP) | - | - |
| Günzel-Apel et al., 1997 | 33 oestrous cycles and nine pregnancies from 31 bitches of five breeds | Samples at: early and late proestrus, ovulation, end of oestrus, days 30, 60 and 90 of metestrus, and anoestrous | Haematocrit Microhaematocrit centrifuge | None stated | Mean ± S.D | Yes | Yes | 45.5±3.3 (NP)  45.0±4.6 (P) | Not stated, shown graphically | 36.3±4.1 (P) | 40.0±2.9 (NP)  35.0±2.4 (P) | - | Not stated, shown graphically |
| Kaneko et al., 1993^†^ | 23 pregnant Beagle bitches | Before pregnancy  Day 55 to 60 of pregnancy | Haematocrit Automatic cytometer | None stated |  | Yes | N/A | - | - | - | - | - | - |
| Tietz et al., 1967 | 22 pregnant, 14 mated not pregnant and 14 not mated Beagle bitches | Weekly samples over an 18-month period including pregnancy | PCV Microhaematocrit | None stated | Mean ± S.D | Yes | Yes | - | 51.0±3.9 (P)  52.0±3.4 (NP)  53.0±2.8 (not bred) | - | 34.0±3.3 (P)  41.0±3.0 (NP)  44.0±3.5 (not bred) | - | - |

^†^ Mean PCV values for bitches were not presented within the published studies. ‡10 pregnant bitches had three samples at oestrus, 4th and last weeks of pregnancy. remaining values for proestrus/oestrus, dioestrus and anoestrus were from single measurements from individual bitches. § The exact stage at which PCV was measured was not stated and was described as ‘pregnant’. ¶ Haematology results for anoestrous (n=34), proestrus (n=3), oestrus (n=4) and immature (n=7) bitches were grouped to compare to PCV values for dioestrus bitches.

References that do not appear in the main manuscript:

Kimberely, E. T., Casal, M. L., O'Donnell, P. A., et al. (2006) Effects of pregnancy on complete blood cell counts and serum biochemical profiles in dogs. Proceedings of the Annual Conference of the Society for Theriogenology. *Theriogenology* 66, 670.
